# Using real-time data to guide decision-making during an influenza pandemic: A modelling analysis

**DOI:** 10.1371/journal.pcbi.1010893

**Published:** 2023-02-27

**Authors:** David J. Haw, Matthew Biggerstaff, Pragati Prasad, Joseph Walker, Bryan Grenfell, Nimalan Arinaminpathy

**Affiliations:** 1 MRC Centre for Global Infectious Disease Analysis, Department of Infectious Disease Epidemiology, School of Public Health, Imperial College London, United Kingdom; 2 Influenza Division, National Center for Immunization and Respiratory Diseases, Centers for Disease Control and Prevention, Atlanta, Georgia, United States of America; 3 Department of Epidemiology of Microbial Diseases, Yale University, New Haven, Connecticut, United States of America; 4 Department of Ecology and Evolutionary Biology, Princeton University, Princeton, New Jersey, United States of America; National Institutes of Health, UNITED STATES

## Abstract

Influenza pandemics typically occur in multiple waves of infection, often associated with initial emergence of a novel virus, followed (in temperate regions) by a resurgence accompanying the onset of the annual influenza season. Here, we examined whether data collected from an initial pandemic wave could be informative, for the need to implement non-pharmaceutical measures in any resurgent wave. Drawing from the 2009 H1N1 pandemic in 10 states in the USA, we calibrated simple mathematical models of influenza transmission dynamics to data for laboratory confirmed hospitalisations during the initial ‘spring’ wave. We then projected pandemic outcomes (cumulative hospitalisations) during the fall wave, and compared these projections with data. Model results showed reasonable agreement for all states that reported a substantial number of cases in the spring wave. Using this model we propose a probabilistic decision framework that can be used to determine the need for preemptive measures such as postponing school openings, in advance of a fall wave. This work illustrates how model-based evidence synthesis, in real-time during an early pandemic wave, could be used to inform timely decisions for pandemic response.

## Author summary

Recent events have made clear the importance of pandemic preparedness. When faced with an initial wave of pandemic influenza, we offer a methodology for decision making with respect to non-pharmaceutical interventions in order to mitigate subsequent waves. For example, delayed school openings during vaccine roll-out can substantially reduce the number of hospitalisations and deaths in subsequent waves. We use first-wave data to quantify future risk and aid the associated policy decisions.

## Introduction

As the recent coronavirus pandemic caused morbidity, mortality and societal disruption on a global scale, the threat of similar disruptions from pandemic influenza remains [1, 2]. The 1918 H1N1 pandemic caused an estimated 50 million deaths worldwide, with a similar case fatality rate to COVID-19 [3, 4]. Today, highly pathogenic strains of avian influenza continue to cause sporadic infections in humans, with occasional instances of human-to-human transmission [5]: preparedness for a future influenza pandemic thus remains as important as ever.

A marked feature of influenza epidemiology is its pronounced seasonality, with already-established influenza viruses causing epidemics each winter in temperate regions of the world. Such seasonality is likely to arise from a combination of factors, and has been associated with environmental conditions including absolute humidity [6], as well as increased transmission amongst schoolchildren with the post-holiday opening of school terms [7, 8]. These seasonal drivers have played a strong role in shaping the dynamics of pandemic, as well as seasonal, influenza. For example, in the USA in 2009, the novel H1N1 virus caused a ‘spring wave’ from April to July, during which an estimated 1.8m-5.7m people experienced symptomatic infection and 9,000–21,000 were hospitalised [9]. The subsequent onset of the influenza season in October 2009 was accompanied by a strong resurgence of the virus (the ‘fall wave’), resulting an estimated 60.8m total infections and 274,304 hospitalisations by April 2010 [10]. Similar multi-wave behaviour emerged in other temperate countries, with the UK experiencing three successive waves [11, 12]. If novel influenza viruses can emerge at any time of year in temperate countries, it is more likely than not that they would do so outside the normal influenza season, which typically spans a 4 month period (late October to late February) in the Northern Hemisphere.

For any future influenza pandemic, population surveillance data collected from any initial, out-of-season wave could therefore give important information for characterising severity and transmissibility. Could such data be used to predict the potential health impact of a subsequent fall/winter wave? If so, could such projections be used in real time, to trigger preemptive control measures in advance of that wave? In this work we addressed these questions using a mathematical model of influenza transmission dynamics, with a focus on pandemic spread in the USA. We present examples of how this framework can be used to guide real-time decision-making for future pandemics.

## The Model

### Model equations

We used a deterministic SIR (susceptible-infectious-removed) model of influenza transmission defined as follows:

$$\lambda_{i}=\beta [1+\phi cos(2\pi \frac{t-t_{lag}}{365})]\sum_{j}C_{ij}\frac{I_{j}^{S}+rI_{j}^{A}}{N_{j}}$$ (1)

$${\dot{S}}_{i}=-\left[\lambda_{i}+v_{i}\left(t\right)\right]S_{i}$$ (2)

$${\dot{S}}_{i}^{V}=v_{i}\left(t\right)S_{i}-\left[\lambda_{i}+u\right]S_{i}^{V}$$ (3)

$${\dot{S}}_{i}^{VP}=uS_{i}^{V}-\left(1-x_{i}\right)S_{i}^{VP}\lambda$$ (4)

$${\dot{I}}_{i}^{S}=\rho \lambda_{i}[S_{i}+S_{i}^{V}+\left(1-x_{i}\right)S_{i}^{VP}]-\gamma I_{i}^{S}$$ (5)

$${\dot{I}}_{i}^{A}=\left(1-\rho \right)\lambda_{i}[S_{i}+S_{i}^{V}+\left(1-x_{i}\right)S_{i}^{VP}]-\gamma I_{i}^{A}$$ (6)

$${\dot{R}}_{i}=\gamma (I_{i}^{S}+I_{i}^{A})$$ (7)

where *S*^*V*^ denotes vaccinated susceptibles, *S*^*VP*^ vaccinated susceptibles with active vaccine protection, *I*^*S*^ symptomatic infectious, *I*^*A*^ asymptomatic infectious, *R* the recovered population, *N* the total population, and the subscripts *i* and *j* index 5 age groups (0–4, 5–17, 18–49, 50–64 and 65+). The contact matrix *C* was taken from [13]. The parameter *ρ* denotes the proportion of cases that are symptomatic, *r* the reduction in infectiousness of asymptomatic cases, *v*_*i*_(*t*) the vaccination rate in age-group *i*, and *x*_*i*_ the corresponding infection-blocking efficacy. To model the effect of vaccination we used the vaccination uptake and efficacy observed in 2009, which began in early October (MMWR week 39, S4 Fig). We assumed a mean delay of 14 days (*u* = 1/14) from administration of vaccine to full protection. For each FluSurv-NET location, we simulated using the corresponding age-stratified sample location populations *N*_*i*_. Hospitalisations were modelled as a multiplicative factor of symptomatic incidence, using the case-hospitalisation ratios given in [14]. Vaccination rates and hospitalisation multipliers were available at national level only, and assumed that these same data could be used independently for each of the states in this analysis. The parameter *ϕ* denotes amplitude of seasonal forcing, *γ* = 1/*T*_*R*_ the recovery rate (where *T*_*R*_ is the mean infectious period), and transmissibility *β* was calculated via the next-generation eigenvalue method in order to yield the desired basic reproductive number *R*_0_.

### Model calibration

**Fig 1:**
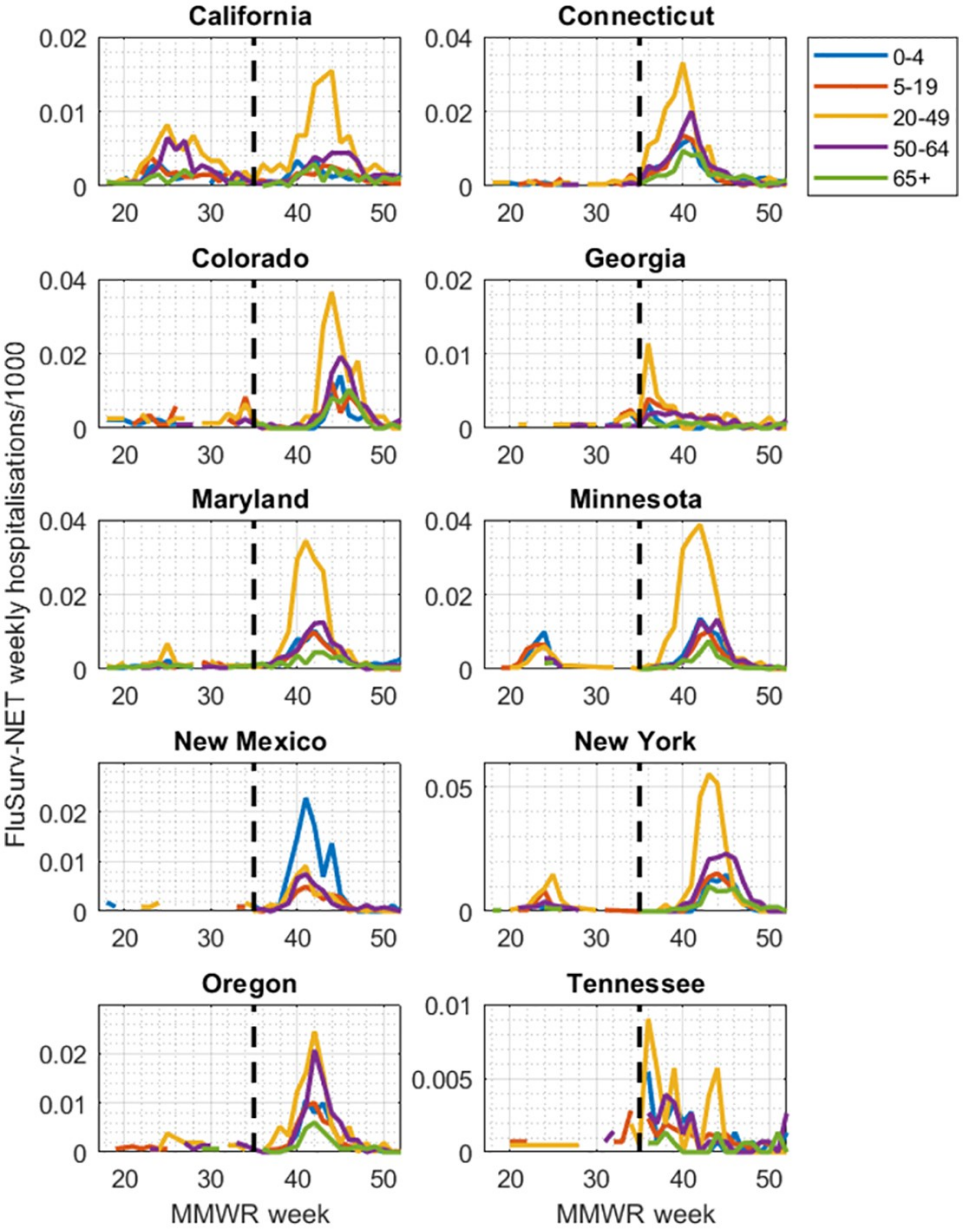
FluSurv-NET data from the 2009 H1N1 pandemic in the USA. Shown is weekly, age-specific data collected by the US Centers for Disease Control and Prevention (CDC), for hospitalisations that were laboratory confirmed as being pandemic H1N1. Each colour denotes a different age group, as indicated by the legend. Panels show data from the different states reporting FluSurv-NET data. Weeks are numbers along the x-axis according to MMWR numbering with, for example, week 35 corresponding to the week beginning on Sunday 30th August. Note that the *y*-axis varies between states. As described in the main text, several of these states (e.g. California) show clear signs of distinct spring and fall waves. For the purpose of the model, we used these data in combination with CDC estimates for the proportion of symptomatic cases that are hospitalised, laboratory tested, and reported through this dataset.

Fig 1 shows FluSurv-NET data during the spring and fall waves of the 2009 pandemic: laboratory confirmed hospitalisations with pH1N1 among children and adults through a network of acute care hospitals in 10 states of the USA [15].

In the model calibration, uncertainty in disease incidence was captured via uncertainty in case-hospitalisation multipliers. We constructed likelihoods for these multipliers using normal distributions for each age group *i*, with mean *μ*_*i*_ and standard deviation *σ*_*i*_, given in S1 Table [14]. For a given parameter set *θ*, our model produces simulated weekly incidence **y**(*t*), desegregated by age group. We then divided these by the corresponding hospitalisation data (shown in Fig 1) to yield simulated case-to-hospitalisation ratios. The overall likelihood L(θ) is then a product of normal likelihoods *N*(*μ*_*i*_, *σ*_*i*_) over all age groups and time points (in practice, we computed the sum of log-likelihoods in calibration).

We performed a Bayesian calibration to the first wave only (up to 1st September, MMWR week 35) using an adaptive MCMC algorithm [16]. We allowed for uncertainty in the coefficients *C*_*ij*_ and accounted for school closures throughout this period by subtracting fixed values *δ*_1_ = 1.41 and *δ*_2_ = 6.17 from *C*_11_ and *C*_22_ respectively, determined by the education-specific contact rates given in [17]. “School closures” thus refers to changes in contact rates in both the 0–4 and 5–19 age groups. Other calibrated parameters were: seasonal amplitude *ϕ*, seasonal lag *t*_*lag*_, proportion of over-52-year-olds with pre-existing immunity, proportion of cases *ρ* that are symptomatic, relative infectiousness of asymptomatics *r*, seed time *t*_0_, basic reproductive number *R*_0_ and recovery rate *γ*. We assumed pre-existing immunity in only those over 52 in 2009 as this is the cohort born prior to the H2N2 pandemic in 1957, when seasonal flu was predominantly of the 1918 H1N1 lineage from which historic infections offered immunity to the 2009 pandemic strain. Since age our age groups was ages 50–64, we assumed that pre-existing immunity applies to the fraction 13/15 of this group, i.e. assuming uniform distribution of ages within the group. A complete set of fitted model parameters and ranges of uniform priors is given in S2 Table. Sampling from the posterior density from the first wave, we projected from 1st September, imposing the change in contact rates due to school openings. Together with seasonality, parametrised by *ϕ* and *t*_*lag*_, this generated a second wave of infection.

A delay in school openings was modelled via a delay in the change in contact rates. The probabilistic risk scores for a given state and delay in school opening were calculated by projecting the corresponding epidemic scenario for each parameter set in our posterior sample. Each simulation yielded a total number of hospitalisations, which were always counted from the week of 1st September, irrespective of delay in school openings.

## Model results

Fig 1 illustrates the difference in size between the two waves. Notably, the states of Georgia, New Mexico and Tennessee reported only sparse data in the spring/summer period: these same states also showed substantially fewer cases being reported in the fall wave per capita, when compared to other states.

**Fig 2:**
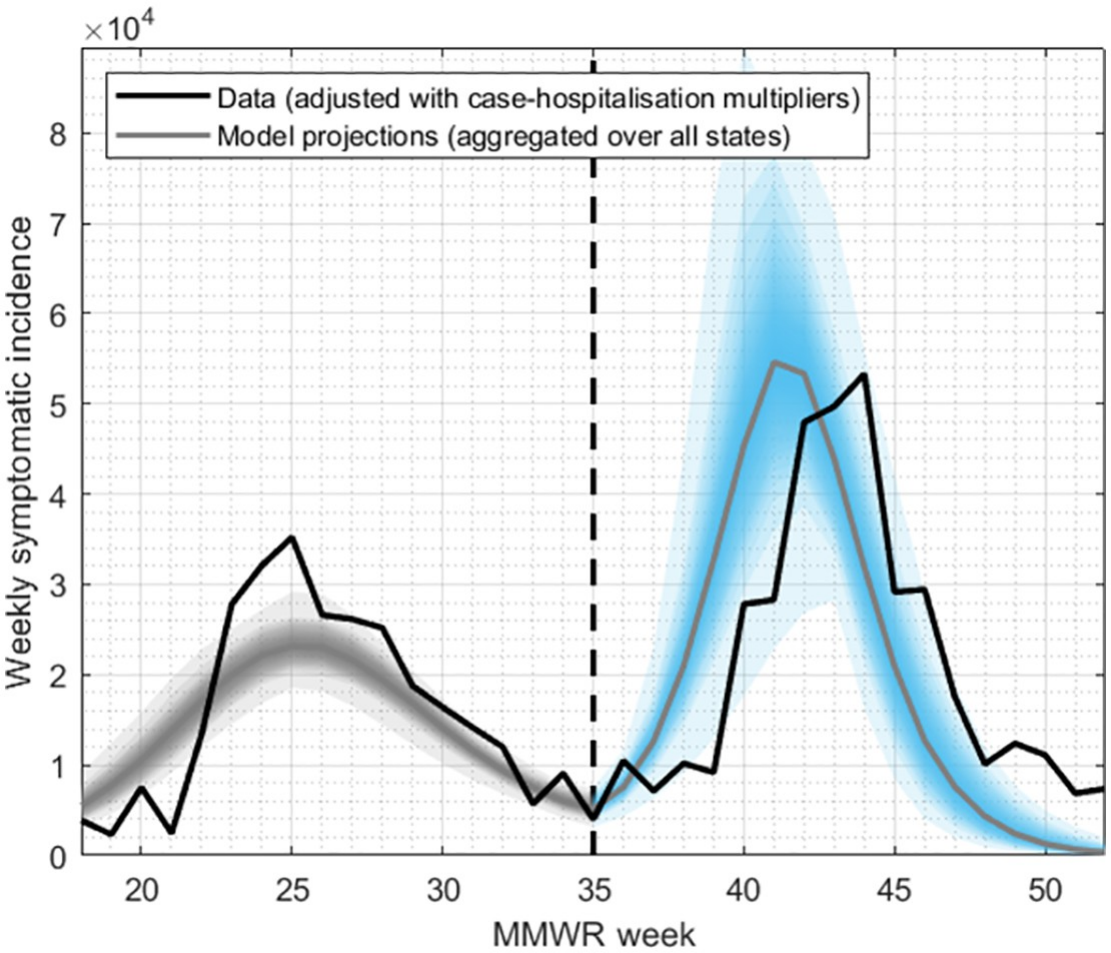
Illustration of the modelling approach, and of model projections, in the example of California. For each state shown in Fig 1, we calibrated the model to the epidemic data from the spring wave (black line, to the left of the vertical dashed line, with aggregated model projections shown in grey shaded area). Using this calibrated model, we projected simulations forward into the fall, taking account of the effect of school openings and environmental forcing (blue shaded area). Although the model projection for epidemic peak timing varied in accuracy across states, our subsequent analysis concentrates on cumulative burden (area under the curve). See S2 Fig for results for other states.

Fig 2 shows results of calibrating the model to the spring wave of the pandemic in California (grey shaded area), and then projecting forward to simulate the fall wave (blue shaded area).

S2 Fig shows outputs disaggregated by state. S3(A) and S3(B) Fig illustrate the performance of the Bayesian Markov Chain Monte Carlo used to perform these calibrations. Although the model tends to estimate fall wave peak timing earlier than actually occurred in the data (week 41 vs week 42 for aggregate results, week 40 vs week 44 for Connecticut), there was reasonable agreement for the size of the fall wave (comparing areas under the curve for model projections and data for incidence). For the remainder of this analysis, we focused on the cumulative burden in the fall wave rather than peak timing.

**Fig 3:**
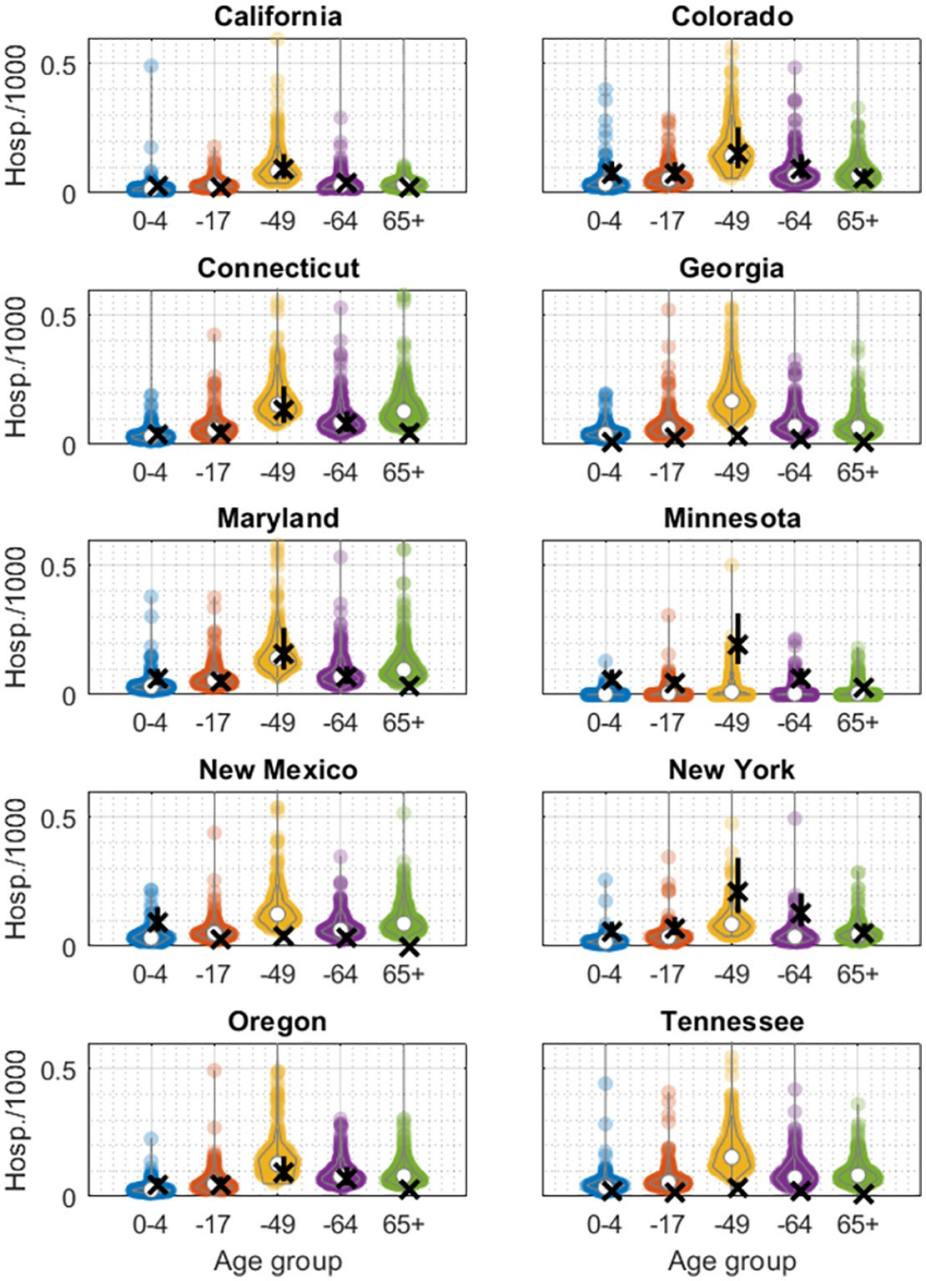
Model projections for cumulative hospitalisations in the fall wave. Each panel shows a different state. Crosses in black show data, vertical black lines show the 90% range of hospitalisation multipliers as given in [14], and coloured points show model-based projections, with each point representing the result of a single sample from the posterior density.

Fig 3 shows a state-wise disaggregation for the cumulative projected hospitalisations in the fall wave. Model projections show good agreement in 7 of the 10 states studied. However, model projections appear less accurate in Georgia, New Mexico and Tennessee, where the model substantially overestimates cumulative hospitalisations in the fall wave. As noted above, these are the states with only sparse data reported in both spring and fall waves.

For those states showing reasonable model performance, we next examined how this framework could be operationalised, to trigger preemptive interventions in advance of the fall wave. Such interventions could involve, for example: physical distancing orders; preemptive school closures; or other non-pharmaceutical measures aimed at reducing opportunities for transmission. As such measures are typically costly and disruptive, any decision to implement them must carefully balance these disruptions against the risks of morbidity and mortality.

## Application

**Fig 4:**
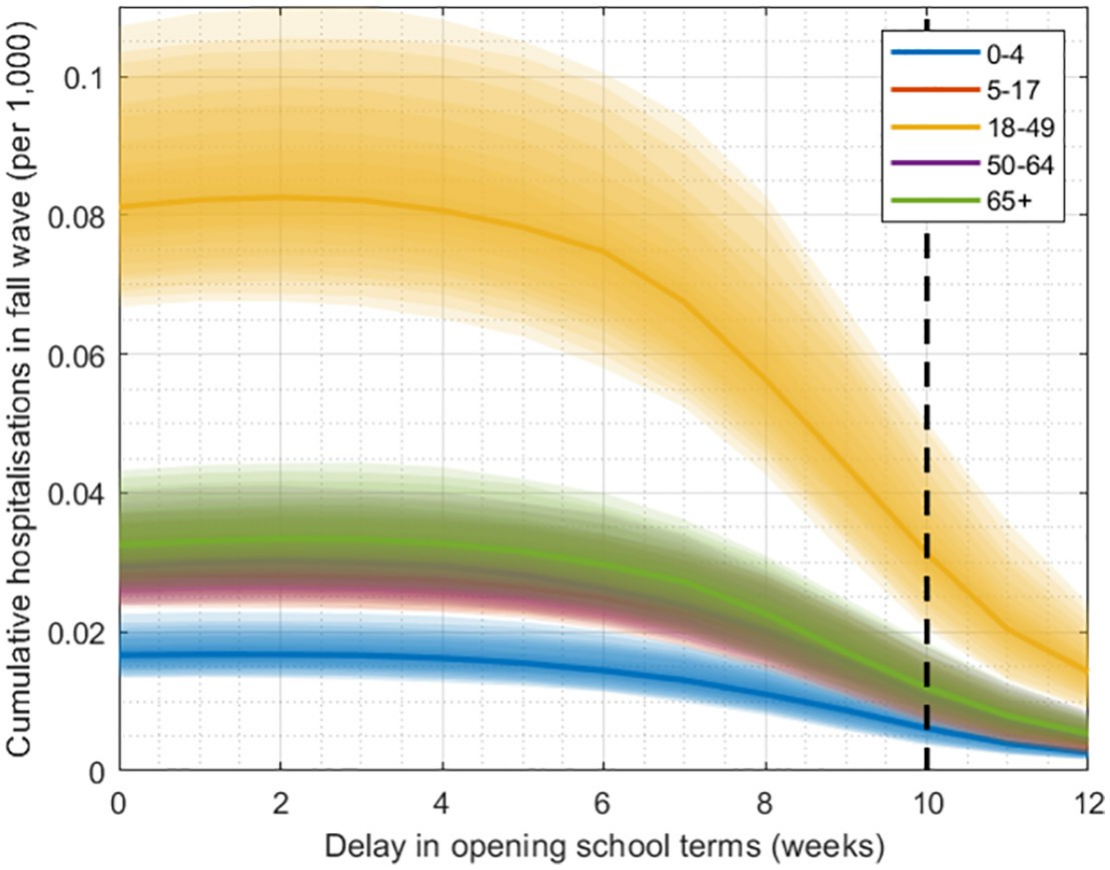
Projected hospitalisations (California) with delayed school openings, assuming the same efficacy and timing of vaccine rollout as occurred in 2009. Each colour shows a different age group as indicated by the legend, while shaded areas show 25–75th percentiles, with 2009 vaccination coverage/efficacy. The vertical dashed line represents the candidate delay of 10 weeks used to illustrate our decision framework in Fig 5.

As an illustrative example in the current analysis, we concentrated on preemptive school closures (i.e. postponing the start of the school term), until a vaccine becomes available. Given the model’s poor performance in predicting second-wave timing, and its better performance in predicting cumulative hospitalizations, we focused on the latter outcome as a criterion for judging whether to implement preemptive school closures. To inform our assumptions for vaccine roll-out, we assumed the same trajectory as in the H1N1 pandemic, when a vaccination programme was initiated in October, ultimately to cover over 25% of the population (see S4 Fig). In a hypothetical scenario where preemptive school closures are implemented for a 2009-like pandemic, Fig 4 illustrates the reduction in fall wave burden, as a function of different durations of intervention. The figure illustrates, for example, that an example intervention of preemptive school closures for 10 weeks, together with the impact of the vaccine rollout, could reduce cumulative hospitalisations in the fall wave by 72% (95% CrI 38–90%).

**Fig 5:**
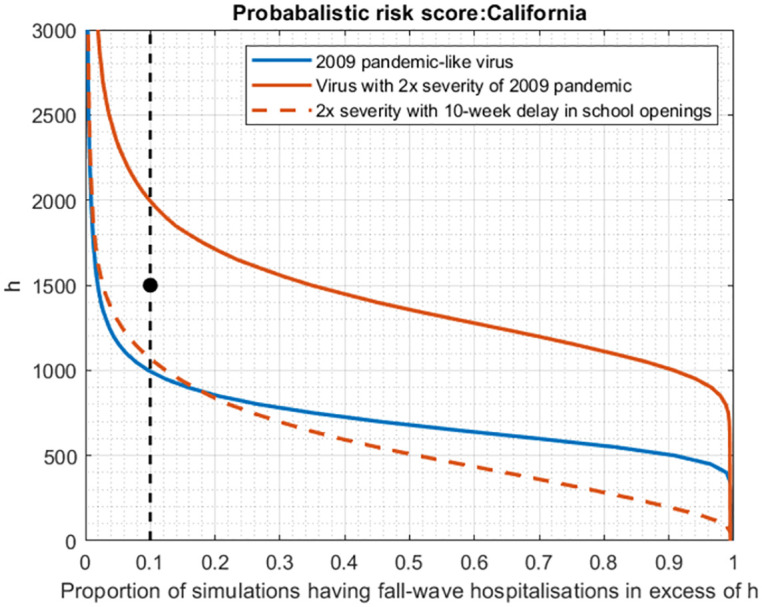
Proposed decision framework for triggering preemptive non-pharmaceutical interventions (NPIs), in advance of the fall wave. Shown, for illustration, is the example of California, and a proposed scenario in which school opening is postponed by 10 weeks. These plots can be interpreted as cumulative probability distributions, for the total hospitalisations projected in the fall wave. As described in the main text, we define a ‘probabilistic risk score’ (PRS) as the probability that fall wave hospitalisations will increase a given threshold, *h*. We assume that preemptive interventions would be triggered if PRS exceeds some threshold probability *P*, with both H and P determined by a policymaker. The figure shows an illustrative scenario where *h* = 1, 500 cumulative hospitalisations, and *P* = 0.1 (‘reference point’, shown as a black dot). Any model-based projections can be represented as a downward-sloping curve on this plot: preemptive interventions would be triggered if the curve intersects the vertical, dashed line at any point above the reference point. As examples, the blue curve shows model projections for a 2009-pandemic-like virus in California (i.e. corresponding to Fig 3A), a scenario that would not trigger preemptive interventions. The solid red curve shows an alternative scenario, of a virus that is equally infectious, but twice as severe (i.e. having twice the risk of hospitalisation given infection). Such a virus would trigger preemptive interventions; the dashed red curve shows the reduction in hospitalisation risk that would occur, in a scenario where school opening is postponed for 10 weeks until vaccine rollout is underway (assuming the same vaccine introduction and rollout scenario as occurred in 2009–2010, in response to the pandemic).

As a decision tool for when to trigger such measures, we defined the ‘probabilistic risk score’ (PRS) as the probability that cumulative hospitalisations in the fall wave will exceed a threshold of *h* per capita. We assumed that this risk score would be evaluated at the end of the spring wave, and that preemptive school closures would be triggered if PRS exceeds a given threshold *p*. In practice, both *h* and *p* would be determined by a policymaker, and can be interpreted as reflecting considerations of healthcare capacity (cumulative hospitalisations, *h*), alongside tolerance of uncertainty (*p*). As an illustrative example, we assumed a scenario where *h* = 1, 500 and *p* = 0.1. Fig 5 illustrates this decision tool being applied to the 2009 H1N1 pandemic in California, as well as an alternative scenario with a hypothetical virus having greater clinical severity (the same infectivity, but twice the risk of hospitalisation). Under these parameters, a 2009-like virus would not trigger the intervention (blue line), whereas a more severe virus would do so (solid red line). The FluSurv-NET data set estimates 1, 013 hospitalisations in California during the second wave, consistent with our decision not to trigger an intervention. Moreover in the second (more severe) scenario, preemptive school closures for 10 weeks would bring PRS substantially below the threshold (dashed red line).

## Discussion

In any future influenza pandemic, early and accurate information will be critical in deciding how best to respond. Here we have examined how mathematical modelling of transmission dynamics could be used to analyse surveillance data in the early stages of a pandemic, to inform decisions for preemptive non-pharmaceutical interventions, in advance of any second wave of infection. Given that our approach is based on a relatively simple compartmental model of influenza transmission, it is perhaps unsurprising that this model cannot fully capture the dynamics of the second wave: importantly, in many states the model poorly captures the correct timing of the fall wave peak (S2 Fig). Nonetheless, for states where there is sufficient data, this simple model shows reasonable projections for cumulative hospitalizations in the fall wave (Fig 3). Thus, our work illustrates how simple modelling approaches could be used to inform decisions for pre-emptive public health measures aimed at reducing cumulative hospitalisations (Fig 4).

Different model approaches may be better suited for different objectives. For example, the peak timing of any severe pandemic wave is arguably as important for health system preparedness as cumulative burden: in practice, public health authorities may be as concerned with postponing, as well as ‘flattening’, the epidemic curve. Factors driving influenza transmission are complex and multifactorial [18], and thus challenging to model predictively in a mechanistic way. To better capture epidemic timing, alternative approaches might include recognised drivers such as absolute humidity [19, 20]. However, any resulting improvements in a model’s predictive power should be weighed against the increased model complexity that arises as a result. A key advantage of the simple framework that we have demonstrated here is that it can be readily deployed in real time. In the example of absolute humidity, there will be a need to supply the model with accurate, medium-term projections for this indicator, perhaps necessitating integration with weather and climate models developed for this purpose [21].

Our modelling approach performs less well in states having only sparse data for the spring wave (Figs 1 and 3). Such sparsity could be explained either by under-reporting, or by a genuinely lower level of influenza activity in these locations. However, it is notable that these states also reported systematically low numbers in the fall wave as well (Fig 3 and S1 Fig), suggesting that data reporting or underestimating relative to other sites may be driving factors. We focused on data for laboratory confirmed hospitalisation because it was the least affected by changes in surveillance practices or other circulating pathogens during the pandemic [22]. Nonetheless, an important area for future work is to explore the potential for incorporating other forms of data as well, including syndromic and virological surveillance collected from the primary care level and above [23]. Combining different streams of data in this way could offer a helpful approach, to compensate for shortfalls in any individual data stream. Moreover, model calibration to an unmitigated phase allows us to capture important epidemiological properties of an outbreak, most crucially the basic reproductive number. Though such calibration does not require an entire wave to be unmitigated, any changes in contact patterns during the calibration period is likely to bring additional uncertainty into the model. Similarly, in scenarios where the vaccination programme or other mitigating measures are implemented between the first and second waves, it will be important to reflect these interventions appropriately, in the model calibration and projections. Again, given the difficulty of estimating intervention effectiveness at such an early stage in the pandemic, incorporating these factors is likely to bring additional uncertainty into the model.

As an example of non-pharmaceutical interventions, we have modelled preemptive school closures, i.e. postponing the opening of schools. We note that our estimates for the impact of these measures are driven by modelled variations in the age-specific contact matrix, depending on whether schools are in or out of session. In turn, these variations are derived from estimated contact rates in an education setting [17]. In future, such estimates would benefit from primary evidence for the ‘real world’ impact that could arise from preemptive school closures. Because much of the available, primary evidence arises from closures that occurred during the course of an epidemic, it is likely to underestimate the impact of preemptive measures. The absence of influenza cases in 2020–2021 suggests that the combination of non-pharmaceutical interventions employed to mitigate the spread of COVID-19 prevented major influenza outbreaks, though school closures were only a part of this effort [24]. Available evidence for the impact of school closures in the mitigation of COVID-19 remains equivocal, and the apparently milder natural history of infection in children, in comparison with influenza, may limit the generalizibility of this specific intervention. Nonetheless, other statistical analysis, taking advantage of country-level variations in school opening dates, suggests that later school openings were indeed associated with reduced epidemic peaks in the fall of 2009 [25].

It is also important to note that, because our simple model framework does not perform optimally in capturing epidemic timing, it may also be biased in its projections of interventions linked to specific calendar times. In particular, a vaccination drive that is initiated early with respect to an epidemic will generally achieve greater impact than one that is initiated late. Our model is therefore likely to underestimate the impact of vaccination in states where its projected second-wave timing is earlier than in reality (e.g. California), and vice versa in states where it is later than in reality (e.g. Colorado). Understanding potential biases such as these will help in interpreting model findings, for decision-making.

Our analysis has other limitations to note. Our mathematical model involves several simplifications: averaging at the state level, it does not address the marked intra-state, spatial heterogeneity seen in the 2009 pandemic [7], indeed heterogeneity that is likely to be displayed by any future pandemic as well. Further work could seek to address these complexities by incorporating spatial structure. However, it would be important for any such approach to maintain a balance between complexity, and rapid deployability, during a pandemic. Additionally, our estimates depend on case-to-hospitalisation multipliers, which were estimated during the course of the 2009 pandemic by combining careseeking interviews with other sources of evidence [7]. In any future pandemic, an application of this approach would likewise necessitate such evidence generation, which would need to be implemented rapidly, during the few months of the initial wave. Alternatively, establishing readiness for serological surveillance [26] would likewise provide critical information during the course of a future pandemic, to help translate hospitalisation and syndromic surveillance data to actual burden in the community.

In conclusion, the coronavirus emergency has highlighted the difficult choices faced by decision-makers, in the face of a pandemic: how to weigh the societal disruptions of sweeping interventions against the need for rapid, decisive action to mitigate the spread of a dangerous pathogen. Our work illustrates how simple, rapidly deployable models could contribute towards decision-making, even if focused on specific outcomes such as total hospitalisations. Overall, while surveillance plays a critical role in informing these decisions, dynamical analysis of this data could offer additional, important insights for preemptive action.

## Supporting information

S1 Fig Cumulative hospitalisations by state in the first (left) and second (right) waves of the 2009 influenza pandemic. The second wave is counted from week 35 (inclusive), the week of 1st September.
(TIF)

S2 Fig Model calibrations and projections by state. (TIF)

S3 Fig Marginal densities of the Bayesian fit to first-wave data for California: (A) basic reproductive number *R*_0_ and recovery rate *γ*; (B) *R*_0_ and amplitude of seasonality *ϕ*_2_. (TIF)

S4 Fig 2009 influenza pandemic vaccine roll-out in the USA. (TIF)

S1 Table Mean and standard deviation of multipliers linking symptomatic cases and hospital admissions. (XLSX)

S2 Table Model parameters varied in Bayesian calibration: And boundary values of uniform priors; mean, median and and 95% confidence bounds for each state. (CSV)
