# Supplementary figures and images for: Using real-time data to guide decision-making during an influenza pandemic: A modelling analysis
Source: PLoS Comput Biol. 2023 Feb 27;19(2):e1010893. doi: 10.1371/journal.pcbi.1010893 (PMC9997955; doi:10.1371/journal.pcbi.1010893)

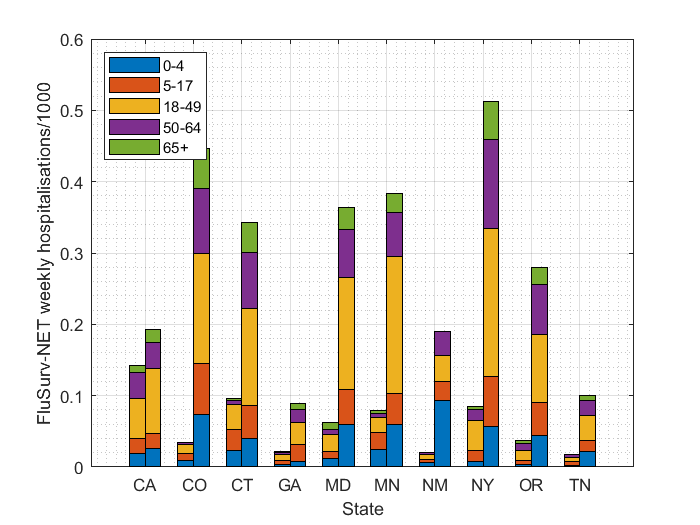

Supplement: S1 Fig — The second wave is counted from week 35 (inclusive), the week of 1st September. (TIF) [file pcbi.1010893.s001.tif]

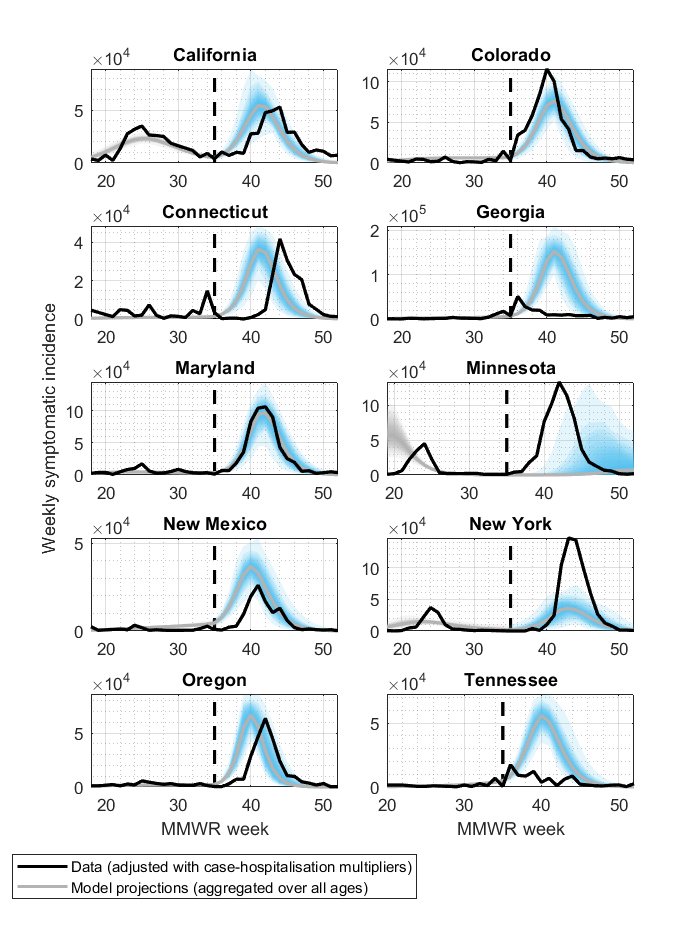

Supplement: S2 Fig — (TIF) [file pcbi.1010893.s002.tif]

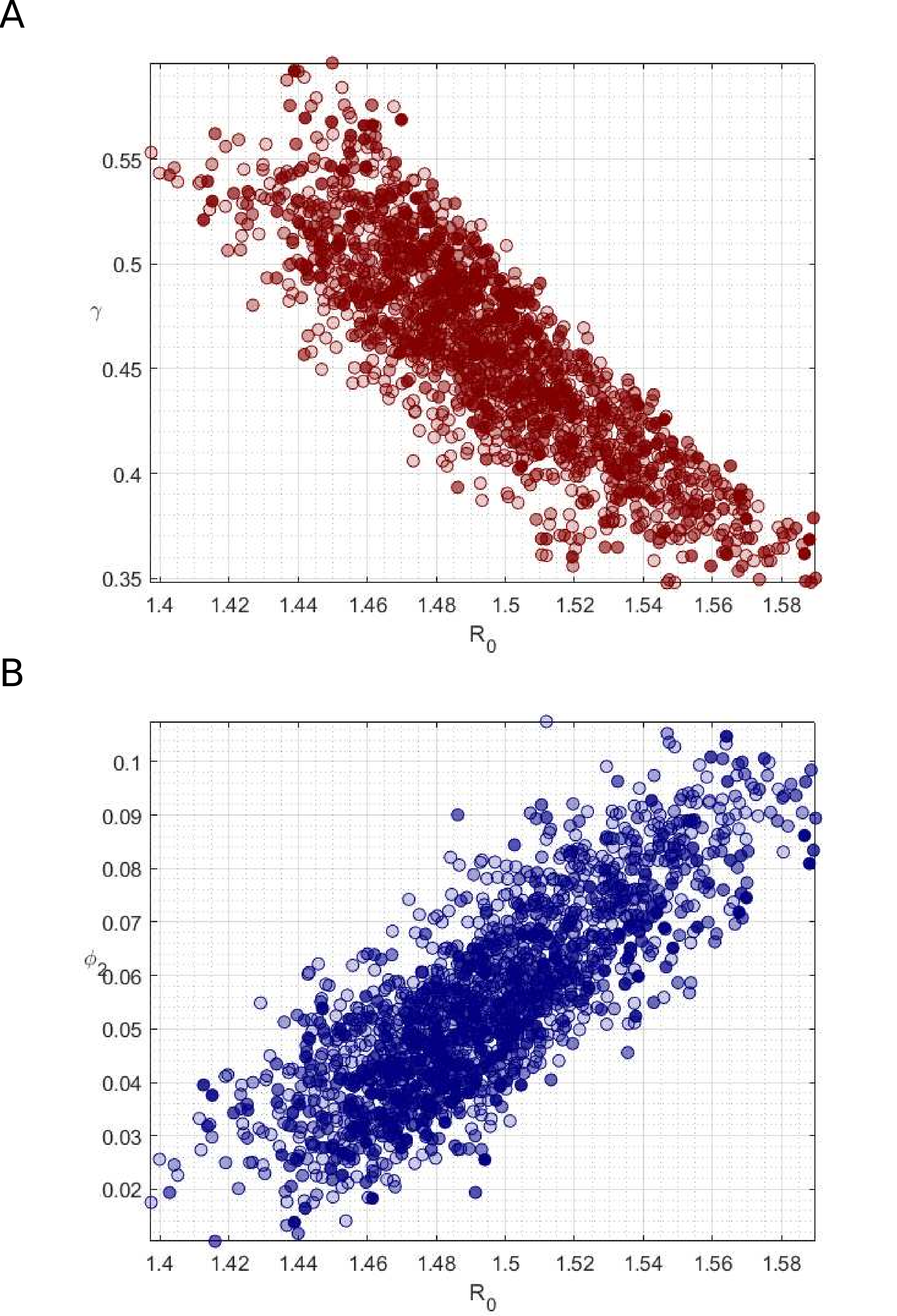

Supplement: S3 Fig — (TIF) [file pcbi.1010893.s003.tif]

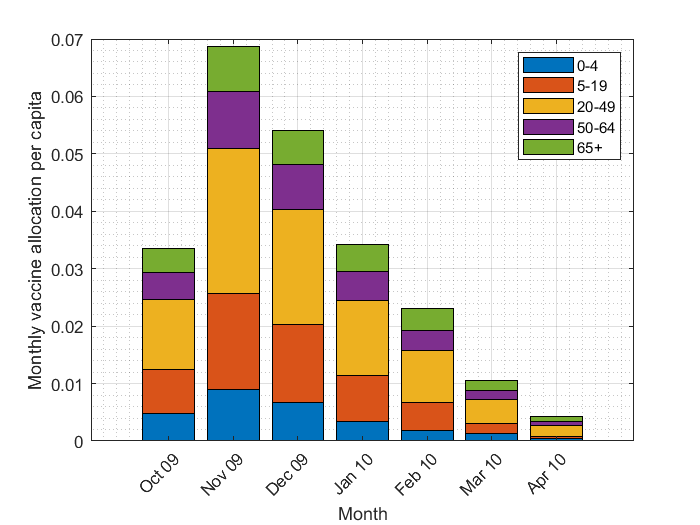

Supplement: S4 Fig — (TIF) [file pcbi.1010893.s004.tif]
